# Expression of circulating oar-miR-485-5p and oar-miR-493-5p during the estrous cycle and early pregnancy in ovine plasma

**DOI:** 10.1590/1984-3143-AR2023-0115

**Published:** 2024-03-04

**Authors:** Eyyup Hakan Ucar, Mustafa Hitit, Mehmet Kose, Mehmet Osman Atli

**Affiliations:** 1 Department of Obstetrics and Gynecology, Faculty of Veterinary Medicine, Aydin Adnan Menderes University, Aydin, Turkey; 2 Department of Animal Genetics, Faculty of Veterinary Medicine, Kastamonu University, Kastamonu, Turkey; 3 College of Agriculture, Food and Natural Resources, Prairie View University, Prairie View, TX, USA; 4 Department of Obstetrics and Gynecology, Faculty of Veterinary Medicine, Dicle University, Diyarbakir, Turkey; 5 Department of Reproduction and Artificial Insemination, Faculty of Veterinary Medicine, Harran University, Sanliurfa, Turkey

**Keywords:** microRNA, plasma, oar-miR-485-5p, oar-miR-493-5p, ovine

## Abstract

In the current study, we aimed to assess the expression levels of two circulating microRNAs (miRNA) (oar-miR-485-5p and oar-miR-493-5p) in the ovine plasma during the peri-implantation. After mating, we collected the plasma samples from a total of 8 ewes on day 22 of pregnancy (P22; n = 4) and day 22 of the estrous cycle (C22; n=4). We used mature miRNA sequences for oar-miR-485-5p and oar-miR-493-5p out of one hundred fifty, which were retrieved from our microarray results of previous study. We showed that the miRNA expression of oar-miR-485-5p and oar-miR-493-5p were upregulated in P22 (P<0.05) when compared to C22. Those two miRNAs targeted 311 target genes in the peri-implantation period of pregnancy. Furthermore, we revealed 151 GO/pathway terms in biological process (BP) and 25 GO/pathway terms in molecular function (MF), while we demonstrated 13 GO/pathway terms in cellular component (CC). We revealed three hub genes as interleukin 2 (IL2), interleukin 18 (IL18), and C-X-C Motif Chemokine Ligand 10 (CXCL10). In conclusion, both miR-485-5p and oar-miR-493-5p have the potential to be a biomarker to understand peri-implantation of the ovine pregnancy in the aspect of pregnancy-reflected changes in maternal plasma.

## Introduction

Interplay between the embryo and the endometrium of the uterus is essential to the development of a healthy pregnancy during the peri-implantation stage of pregnancy (Bazer et al., 2008). Early pregnancy is closely managed at the gene expression level by various substances, which can be implied in the regulation of implantation (Alak et al., 2020); however, the precise functional mechanisms remain unknown. Earlier research has shown that dynamic alterations in the expression of miRNA are involved in the implantation of embryos in many different species (Kose et al., 2022; Stepicheva et al., 2015). In addition to their role in expressed tissues of interest, distinctively in the endometrium (Hume et al., 2023), miRNAs have the ability to be released into the extracellular fluid, thereby establishing cellular interaction and serving as an early marker associated with the specific state of cell functioning (Hitit et al., 2015; Légaré et al., 2022).

Short non-coding RNA molecules known as miRNAs modulate gene expression post-transcriptionally (Bartel, 2004; Hitit et al., 2013). Although miRNAs have a functional role in normal biological processes, abnormal expression of miRNAs is linked with disease state (Bartel, 2004; Vasudevan et al., 2007). RNA polymerase II and III microRNA mediate transcription (Cai et al., 2004), subsequently in the RNA-induced silencing complex (RISC), binds to the 3’ untranslated region (3’-UTR) of mRNA. miRNAs with a global profile and those with differential expression have been demonstrated to be regulated at several stages of the reproductive process, including implantation and placentation (Meng et al., 2021; Shekibi et al., 2022). As a result, miRNAs were shown to be differently expressed during the peri-implantation phase (Chakrabarty et al., 2007). Their expression patterns differ between implanted and non-implanted locations the extracellular vesicle, microRNAs that are either derived from the placenta or the embryo have the potential to enter the bodily fluids (Hitit et al., 2022a; Tan et al., 2020). Because of their sturdy structure, miRNAs are able to withstand damaging effects such as freezing and thawing as well as high temperatures; as a result, using them to identify early pregnancy is the most cutting-edge method currently available (De los Santos Funes et al., 2023). Particularly in domesticated animals, pregnancy-associated plasma samples from cattle, goats and as well as serum samples from sheep have revealed distinct miRNA expression profiles between pregnant and non-pregnant groups. If these unique miRNA expression profiles are indeed signatures of early pregnancy stages, then circulating markers could serve as potential diagnostic tools (Ioannidis and Donadeu, 2016; Zang et al., 2021). In more recent years, it has come to light that EV-associated circulating miRNAs can serve as diagnostic biomarkers for both maternal-fetal communication and pregnancy-related diseases (Hume et al., 2023).

Specifically, we demonstrated that profiling of circulating miRNAs in maternal plasma could be potential biomarkers of early pregnancy (Hitit et al., 2022a) while also some miRNAs had similar expression patterns in ovine endometrium between the estrous cycle (on days 12 and 16) and early pregnancy (on days 12 and 16) (Kose et al., 2022). Therefore, based on our review of this relevant literature, we postulated that potential circulating levels of oar-miR-485-5p and oar-miR-493-5p would also change during the peri-implantation period of ovine pregnancy and that this alteration in plasma expression might function as a potential plasma indicator for understanding the relationship of early fetal and maternal sides interactions. In this study, we aimed to examine the expression of circulating oar-miR-485-5p and oar-miR-493-5p in the plasma during both estrus cycle and early pregnancy in ewes as a consequence.

## Methods

### Experimental method and sample collection

The study was confirmed by International Agricultural Research Institute Ethical Research Committee of Bahri Dagdas for animal experiments and sample collection. We used twenty-four multiparous ewes (3- to 5-year-olds, n=24), which were arbitrarily divided into cyclic (n=4) and pregnant (n=4) groups. Animals were fed to meet the nutritional criteria of the NRC (2007). Throughout the trial, we used supplements (vitamin and mineral with salt mix and marble powder) as needed.

We used two cloprostenol (a synthetic form of prostaglandin F_2_ alpha; PGF_2_α, 125 mcg) injections 11 days apart to synchronize the ewes' estrus cycles (Hitit et al., 2022b). We used teaser rams to confirm estrus shortly after the second injection. Teaser rams were utilized to monitor sheep estrus at eight-hour intervals for five days following the second injection, and ewes that indicated estrus were saved. As a result, the ewes were allowed to finish their cycle, and teaser rams were utilized to record the new natural estrus. The ewes mated (day 0) twice with fertility-proven rams during this imminent estrus. The estrus day was considered as day zero in the cyclic group (day 0). Following mating, we slaughtered ewes on day 22 (post-implantation, n = 4; P22), as well as the corresponding estrous cycle days of 22 (n = 4, C22). During 22 days of pregnancy (pregnancies were confirmed using transrectal ultrasound), we just observed one embryonic trophoblast in the lumen of uterine (Bazer et al., 2012).

### Blood sample processing and extraction of RNA

Prior to slaughtering the sheep, we took blood samples from the vena jugularis into EDTA-treated tubes for plasma extraction. We centrifuged the blood samples for 13 minutes at 1600*g* to separate the plasma, which we then kept at -80^o^C. Ewes with a single corpus luteum on each ovary were used to collect the plasma samples. To thaw the plasma, we used a dry bath set to 20 degrees. Subsequently, 250 ul of plasma was processed through the miRCURY RNA Isolation Kit—Biofluids (Exiqon #300112 Vedbaek- Denmark) in compliant with the manufacturer's instructions to isolate total cell-free RNA (Hitit et al., 2022a). DNA contamination was removed from samples using on-column DNase according to manufacturer's instructions. To that end, we used 50 ul of RNase-free water to elute RNA samples. The miRNA was stored in tubes at -80 °C until the analysis.

### Selection of miRNAs and raw data preparation following microarray assay

We used mature miRNA sequences for oar-miR-485-5p and oar-miR-493-5p out of one hundred fifty, which were retrieved from microarray results. Then the microarray setup was briefly explained. Using the GeneChip miRNA 4.0 Array (Affymetrix, USA), set up to obtain mature miRNA sequences in miRBase (20.0) (http://mirbase.org/ftp.shtml), the miRNA profile from ovine plasma samples were examined. Affymetrix GeneChip Command Console software was used to compute the signal from the probes as cell intensity files (*CEL files), and Transcriptome Analysis Console software was used to examine the results. We compared samples through fold-change with an independent t-test between the pregnant and non-pregnant. Consequently, differentially expressed miRNAs was evaluated using p-value through the Benjamini-Hochberg algorithm. R (v. 3.1.2.).

### Target gene prediction of oar-miR-485-5p and oar-miR-493-5p

The miRNAconsTarget online tool through sRNAtoolbox (http://bioinfo5.ugr.es/srnatoolbox) was used to predict oar-miR-485-5p and oar-miR-493-5p target genes in plasma samples. The provided input data is assessed with independent prediction using animal-based techniques. TargetSpy, miRanda (pairing score > 150 and energy score <-15), and PITA (energy score <-15) are the three prediction algorithms. A possible miRNA target was identified as the common target gene suggested by all three techniques.

### Protein-Protein interaction (PPI) network and hub gene selection from target genes

The STRING database (version 11.5, http://string-db.org) was used to create the functional network association between the target genes, which was then displayed in Cytoscape (version 3.9.0). The target gene PPI network was conveyed and then evaluated in Cytoscape. We used CytoScape plugin CytoHubb for the determination of significant nodes by mixing different topological calculations, including Maximum neighborhood component (MNC), Maximal cilque centrality (MCC), Degree, EcCentricity (EC), and Edge percolated component (EPC) from PPI network (Chin et al., 2014). We ranked the overlapping genes through these five algorithms that shows the hub genes.

### GO enrichment analysis of predicted target genes

In order to examine the functional enrichment of GO keywords, we employed the program g: profiler (Raudvere et al., 2019). The enrichment analysis's parameters were as follows. We chose a unique organism to represent *Ovis aries* (sheep). Sequential GO analyses were performed on cellular component (CC), molecular function (MF), and biological process (BP) data. The user threshold was set at 0.05 when using the g:SCS method to analyze multiple testing adjustments for P-values based on GO and pathway enrichment analysis.

### RT-qPCR analysis

RT-qPCR was used to examine the universal reverse primer, forward primer, and Stem-Loop primer sequences of oar-miR-485-5p and oar-miR-493-5p (Table 1). We prepared a reverse transcription reaction (USB, Cat no: 75780) using the First-Strand cDNA Synthesis Kit for RT-qPCR. The following were the reverse transcriptase reaction conditions: 34 min at 15°C, 53 min at 45°C, 12 min at 94°C, and 7 min at 4°C. After cDNA synthesis by reverse transcriptase reaction, samples were analyzed by RT-qPCR with VeriQuest Fast SYBR Green RT-qPCR Master Mix (USB, Cat no: 75690). The following conditions were utilized for the RT-qPCR reaction: polymerase for 8 min at 94°C, 42 cycles of denaturation for 30 s at 95°C, annealing for 45 s at 54°C, and extension for 55 s at 72°C, with fluorescence data collection occurring during extension. Using the previously described 2−^ΔCt^ approach, log transformation of the data was conducted (Livak and Schmittgen, 2001). A statistical analysis was accomplished on values adjusted to the reference gene.

**Table 1:** The list of miRNA primers used for gene expressions in RT-qPCR.

**Transcript ID**	**Sequences of forward primer, universal reverse, and stem-loop primers**
**oar-miR- 493-5p**	FP: 5' - TGGTGTTGTACATGGTAGGCT - 3'
	RP: 5' - GTGCAGGGTCCGAGGT - 3'
	S-LP:5'-GTTGGCTCTGGTGCAGGGTCCGAGGTATTCGCACCAGAGCCAACAATGAA- 3'
**oar-miR-485-5p**	FP: 5' - TGTTTTTTAGAGGCTGGCCG - 3'
	RP: 5' - GTGCAGGGTCCGAGGT - 3'
	S-LP: 5' - GTTGGCTCTGGTGCAGGGTCCGAGGTATTCGCACCAGAGCCAACCGAATT- 3'

## Results

### miRNA expression analysis and target prediction, and PPI Network Construction

**Figure 1:**
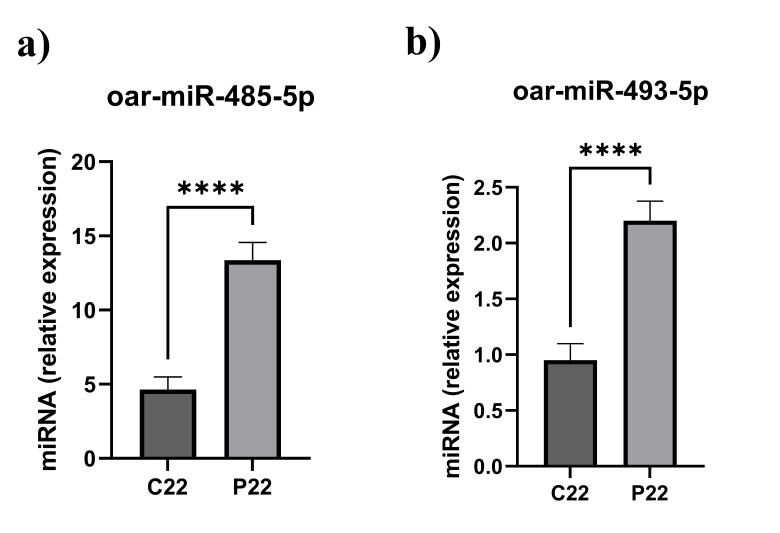
miRNA expression of oar-miR-485-5p and oar-miR-493-5p in RT-qPCR. Expression of (a) oar-miR-485-5p and (b) oar-miR-493-5p. Data are shown as relative abundance ± SEM, P < 0.05; (Estrous cyclic day 22:C22, Pregnant day 22: P22, indicates the group). The 2−ΔΔCt method is used to analyze relative mRNA expression levels. **** denotes; P ≤ 0.0001.

**Figure 2:**
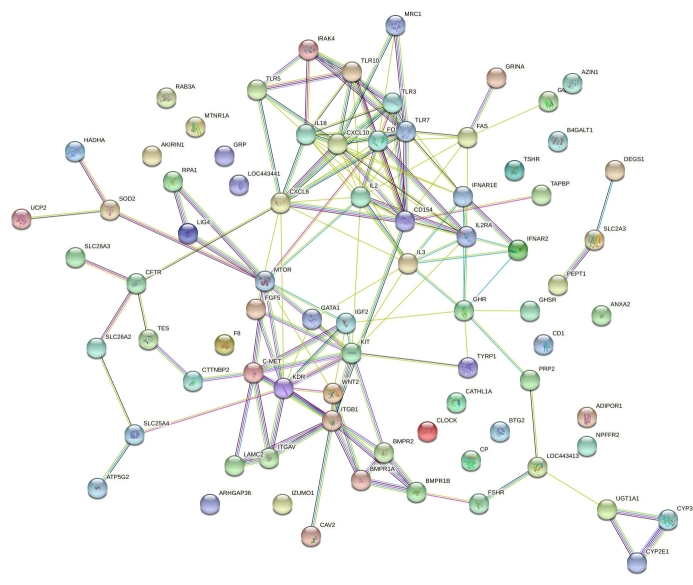
PPI network analysis of predicted target genes in ovine plasma.

In plasma, the miRNA expression of oar-miR-485-5p increased in P22 (*P*<0.05) relating to P22 (Figure 1a). Likewise, the miRNA expression of oar-miR-493-5p in P22 (*P*<0.05) when comparing to P22 (Figure 1b). We predicted target genes of the oar-miR-485-5p and oar-miR-493-5p using the online tool (miRNAconsTarget) through sRNAtoolbox according to animal species-based estimation with three algorithms (TargetSpy, PITA, and miRanda). Two miRNAs targeted 311 target genes between the estrous cycle and pregnancy. We excluded overlapping target genes from cyclic and pregnant, and then 91 with four consensus target prediction algorithms were stored to run STRING online database. Subsequently, 78 nodes and 151 edges from PPI network was submitted to the Cytoscape software (Figure 2).

### Functional interaction network and hub selection of predicted target genes

**Figure 3:**
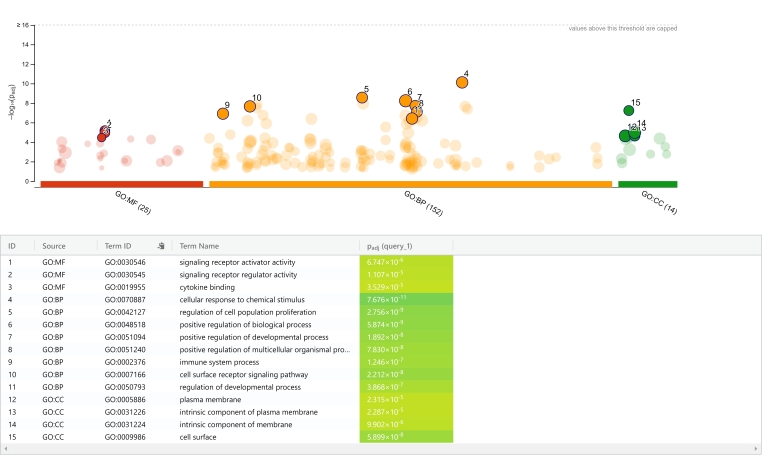
Functional enrichment analysis of target genes in ovine plasma. GO: gene ontology; BP: biological process; CC: cellular component; MF: molecular function.

We revealed 151 GO/pathway terms in BP from the cyclic vs pregnant endometrium, which the most significant terms were (GO:0070887) cellular response to chemical stimulus, (GO:0042127) regulation of cell population proliferation, (GO:0048518) positive regulation of biological process, (GO:0051094) positive regulation of developmental process, (GO:0051240) positive regulation of multicellular organismal process, (GO:0002376) immune system process, (GO:0050793) regulation of developmental process, and (GO:0007166) cell surface receptor signaling pathway (Figure 3). We demonstrated 25 GO/pathway terms in MF, which included (GO:0030546) signaling receptor activator activity, (GO:0030545) signaling receptor regulator activity, and (GO:0019955) cytokine binding (Figure 3). However, in CC, we showed 13 GO/pathway terms linked with (GO:0031226) intrinsic component of plasma membrane, (GO:0005886) plasma membrane, (GO:0009986) cell surface, and (GO:0031224) intrinsic component of membrane (Figure 3). After establishment of PPI networks of predicted target genes, we identified three hub genes between estrus cycle and early pregnancy, by overlapping the top 8 genes according to the five ranked methods in cytoHubba application into Cytoscape. Hub genes were annotated as interleukin 2 (IL2), interleukin 18 (IL18), and C-X-C Motif Chemokine Ligand 10 (CXCL10) (Table 2).

**Table 2:** Hub genes of predicted target genes.

**MCC**	**MNC**	**Degree**	**EPC**	**EcCentricity**
IL18	IL2	IL2	IL2	GHR
CXCL10	IL18	IL18	IL18	IFNAR1E
IL2	CXCL10	CXCL10	CXCL8	IGF2
FOXP3	FOXP3	KIT	CXCL10	IL3
CD154	TLR7	KDR	TLR7	PRP2
TLR7	KIT	CD154	FOXP3	TYRP1
CXCL8	KDR	FOXP3	CD154	GHSR
TLR3	IL2RA	TLR7	IL2RA	IFNAR2

Overlapping hub gene symbols in top 8 from ranked methods. MCC: Maximal cilque centrality; MNC: Maximum neighborhood component; Degree: Node degree; EPC: Edge percolated component; EC: EcCentricity.

## Discussion

Several studies have been used to estimate early pregnancy from livestock animals using conventional methods yet with low success rates. Therefore, filling the gap in the knowledge base on gene expression of circulating miRNA associated with early pregnancy of ewe was the main question of this original study. In this study, we evaluated the expression of two circulating miRNA (oar-miR-485-5p and oar-miR-493-5p) in plasma throughout the critical period of peri-implantation. oar-miR-485-5p and oar-miR-493-5p was evaluated on days of pregnancy (P22) following mating and days of the estrous cycle (C22).

In our study, the target prediction of oar-miR-485-5p and oar-miR-493-5p revealed several GO terms, which ovine circulating miRNAs in plasma may modulate biological and molecular control of gene expression during early pregnancy (Cleys et al., 2014). We indicated that those target genes of GO terms might be implied in early pregnancy, such as (GO:0051240) positive regulation of multicellular organismal process, (GO:0002376) immune system process, (GO:0050793) regulation of developmental process, (GO:0007166) cell surface receptor signaling pathway, and (GO:0030545) signaling receptor regulator activity (Figure 3). Our study demonstrated that circulating miRNAs may be implied in molecular events throughout pregnancy recognition and embryo implantation since they are originated from extracellular vesicles and uterine epithelia in pregnant ewes are, thus miRNA related regulation are implicated in the key pathway in the pregnancy (Burns et al., 2016; Ono et al., 2022).

In recent years, numerous studies that uncovered many miRNAs have identified circulating plasma miRNAs (Hitit et al., 2022a). Villous trophoblast-derived miRNAs are transported through maternal blood in extracellular vesicles or connected to them (Kotlabova et al., 2011; De los Santos Funes et al., 2023). There were 208 miRNAs found in cattle, and comparing plasma samples taken from pregnant and non-pregnant animals, sixteen showed differential expression (Ioannidis and Donadeu, 2016). According to these findings, it may be inferred that plasma miRNAs are circulating and may function as biomarkers for early pregnancy and the estrous cycle (Lim et al., 2021).

On day 16 of gestation in pregnant pigs, circulating miRNAs have been discovered to be expressed either within the endometrium of pregnant pigs (Krawczynski et al., 2015) within the serum of pregnant pigs (Reliszko et al., 2017) on a gestational day 16 of pregnant pigs. It was discovered that the expressions of miR-23b, miR-30d, and miR-379 were greater in exosomes taken from the umbilical vein on gestational day 90 in sheep blood (Cleys et al., 2014). In a very recent study, comparison of Let-7d-5p microRNA expression in pregnant and nonpregnant cows using blood samples revealed linkages of the cellular and molecular interactions between the cow and embryo (De los Santos Funes et al., 2023). We were able to show that the expression levels of oar-miR-485-5p and oar-miR-493-5p were higher in P22 compared to C22. Confounding the results of previous research, we might be able to infer that oar-miR-485-5p and oar-miR-493-5p are distinguishable throughout the early stages of pregnancy.

We demonstrated that oar-miR-485-5p and oar-miR-493-5p targeted 311 genes, shown in common categories relating to (GO:0050793) developmental process and immune system whereby to facilitate conceptus-maternal interface during early pregnancy. Among the hub genes, CXCL10 was a classical type I IFN-stimulated gene and targeted by oar-miR-485-5p and oar-miR-493-5p. Concurrent with our findings during early pregnancy in ovine plasma, we demonstrated that circulating miRNAs regulate the CXCL10 target gene (Hitit et al., 2022a), whichhad greater expression levels from days 14 to 18 in pregnant cows within peripheral blood leukocytes that (Sakumoto et al., 2018) circulating biomarkers may regulate inhibition and activation of immune functions (Dimitriadis et al., 2005). oar-miR-485-5p and oar-miR-493-5p also targeted interleukin family genes, namely interleukin 2 (IL2) and interleukin 18 (IL18), which those receptors may be regulated in pregnancy in regards to conceptus-derived factors (Ka et al., 2018; Ott, 2019).

## Conclusion

For the first time, we employed RT-qPCR to elucidate the expression levels of two circulating miRNAs (oar-miR-485-5p and oar-miR-493-5p 311) in ovine plasma during early pregnancy. We found that anticipated target genes were enriched in the developmental process and (GO:0002684) immune system modulation, implying that circulating miRNAs could be useful markers during early pregnancy.
